# The work design contribution to educational workers' sustainable wellbeing and performance patterns

**DOI:** 10.3389/fpsyg.2022.1020942

**Published:** 2022-11-09

**Authors:** Amalia Raquel Pérez-Nebra, Brenda Soares Viana, Eva Lira, Pilar Martín-Hernandez, María Luisa Gracia-Pérez, Marta Gil-Lacruz

**Affiliations:** ^1^Social Capital and Wellbeing Research Group (BYCS), Department of Psychology and Sociology, University of Zaragoza, Zaragoza, Spain; ^2^Department of Management, University of Brasília, Brasília, Brazil

**Keywords:** work characteristics, work design, wellbeing, happy-productive worker, teachers, elementary school, social support

## Abstract

Brazilian education faces difficulties relating to performance and illness, suggesting that the characteristics of the work can affect both variables. This study aims to describe the work characteristics that increase the odds of having happy–productive patterns in education workers. A total of 4,598 employees of the Secretariat of Education of the Federal District (SEEDF) participated in the research, answering questionnaires about work design (Brazilian version, with 18 factors), wellbeing (containing three factors), and performance. The results showed that task, social, and contextual characteristics increase the probability of being in the happy–productive pattern, and specifically, Social Support, Feedback from Others, Task Significance, Task Identity, and Autonomy, in this order, should be considered for intervention purposes.

## Introduction

One of the United Nations Sustainable Development Goals Is to increase and protect health and wellbeing (Goal 3). Moreover, societies prosper when social capital is high, i.e., when there is a broader and positive social environment and relationship (Diener and Seligman, 2004; Kiss et al., 2014). A key actor in the relationships of society is the elementary school worker, who directly and indirectly serves many families. However, this professional is not always well (experiences wellbeing) and sometimes does not perform well, with clear repercussions for society. Due to these consequences, this research aims to analyze which work characteristics can facilitate patterns of relationship between wellbeing and performance. Diagnosing the work's characteristics will allow organizations to be healthier.

Education is a fundamental right guaranteed by the Brazilian Federal Constitution, and the teacher and other workers in the school are the main facilitators of the learning process and children's care. The performance of these professionals can be measured subjectively, from his/her self-perception, and objectively, through educational indexes [IDEB, SAEB, and PISA, (Brasil, 2021)]. The results of these indexes have not been satisfactory in Brazil, and the schools that make up the Secretary of State for Education have presented difficulties regarding their performance. Contributing to this scenario, the high rate of sickness absenteeism suggests low wellbeing of the educational worker, which, together, leads to an unhappy and unproductive category.

The International Labor Organization (ILO) considers the education profession one of the most stressful, having evident repercussions on physical and mental health and professional performance (Reis et al., 2006). One of the justifications for the lower-than-expected educational rates and the greater rates of illness lies in the relationship between the wellbeing and performance of the actors involved in the educational context, which is not presented in a sustainable manner for both variables. It is necessary to foster a positive synergy, characterized by Peiró et al. (2014) as “sustainable productive wellbeing synergy,” in a practical and manageable way to help education professionals and managers deal with the phenomenon.

For several years, the relationship between wellbeing and performance was grounded in the “Happy–Productive Worker Thesis” (HPWT), which states that workers with higher levels of wellbeing tend to perform better at work compared to workers with lower levels of wellbeing. However, this proposition has some limitations, such as the focus being only on hedonic wellbeing (e.g., “Happy,” which will be discussed below) to the detriment of eudaimonic wellbeing. Another limitation relates to measuring task performance only, i.e., only one of the dimensions analyzed in the concept, and also to the linearity of the relationship between the two variables, by assuming that the happy worker is productive and the productive worker is happy. However, other patterns of relationships may exist, especially those that establish negative and null associations between these two variables (Peiró et al., 2019; Pérez-Nebra et al., 2021a).

In other words, the HPWT does not explain the nuances of the relationship between wellbeing and performance. In order to overcome the limitations of this model, the proposal is to study the relationship between wellbeing and performance by patterns, where positive and negative relationships between the constructs are possible, namely, where the worker can also be “happy–unproductive” or “unhappy–productive.” Peiró and colleagues (Peiró et al., 2019; Pérez-Nebra et al., 2021a) suggest that it is important to consider antagonistic patterns in redesigning the “happy–productive” worker thesis.

**Figure 1:**
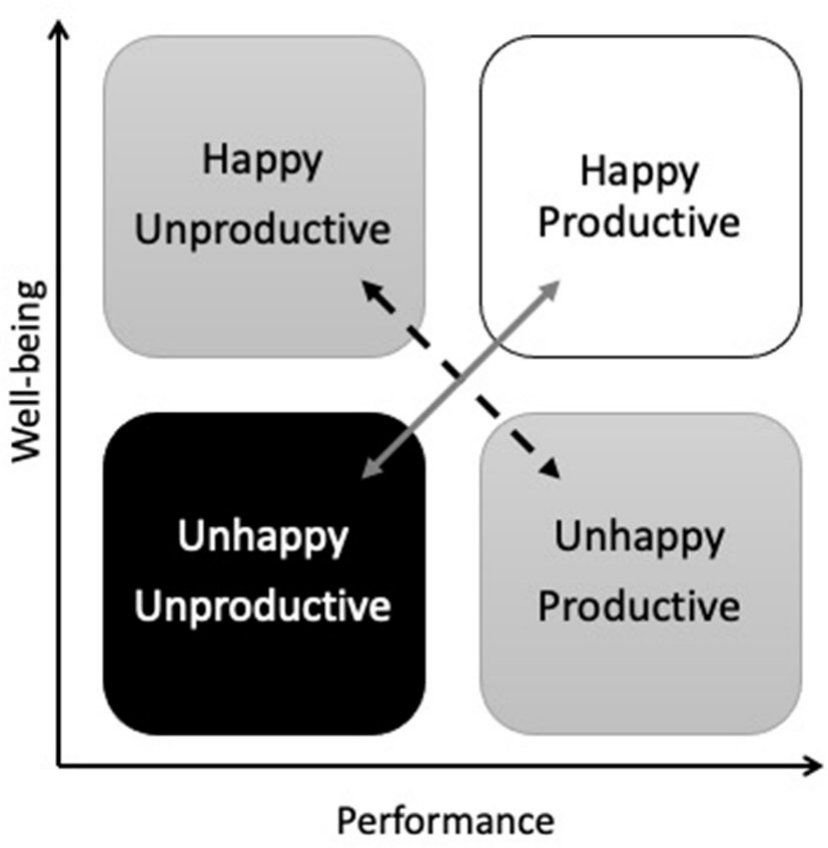
Four basic interactions between wellbeing and performance variables.

In the proposed patterns, four types of interactions between variables are suggested: high wellbeing and high performance, low wellbeing and low performance, high wellbeing and low performance, and low wellbeing and high performance. They can be separated by quadrants, with synergistic relationships such as “happy–productive” or “unhappy–unproductive” and antagonistic relationships such as “happy–unproductive” or “unhappy–productive,” as shown in Figure 1, where the “happy–productive” pattern is the desirable one and interpreted as more sustainable in the long run, unlike the others that can lead to occupational disease (Ayala et al., 2017; Peiró et al., 2014; Peiró et al., 2015; Latorre et al., 2021).

“Happiness” is a generic word that has some limitations. Among them is that it is considered fleeting, only oriented to hedonism, and in this work, it was used to refer to the thesis of the happy–productive worker. Subjective wellbeing is a broad category of a phenomenon that includes different dimensions (Diener et al., 1999). This approach has two predominant perspectives: hedonic and eudaimonic. The former wellbeing is based on the idea of happiness and comprises individual experiences of pleasure and displeasure, which stems from judgments about the positive and negative elements of life (Ryan and Deci, 2001). The eudaimonic covers the individual's self-realization perspective; the achievement of a life's purpose and personal growth are central elements (Ryff and Singer, 2008). Transferring the reading of hedonic and eudaimonic wellbeing to the work context, it can be conceptualized as the prevalence of positive affect and the individual's perception that, in their work, they express and develop their skills and advance in the fulfillment of their life goals (Waterman, 1993; Paschoal and Tamayo, 2008). This triad, between Negative and Positive affect and Fulfillment at Work, will be used in the present work.

The separation into components of wellbeing and its valence (positive and negative) is an attempt to illustrate that some characteristics of work may reduce a Negative Affect, for instance, but not be able to increase the Positive one. Therefore, besides displaying unique relationship patterns with performance, it may also present different antecedents.

Measuring job performance in the educational context is challenging due to the inherent characteristics of educational work. Measuring performance through other people can be problematic because the observer may not have adequate knowledge or because the target behavior depends on several processes that are not fully observable by a single audience (Warr and Nielsen, 2018). Performance in education is not only task-based but also context-based (Pérez-Nebra et al., 2021a). Even with some criticism related to acquiescence, the general self-assessment of professionals is an alternative that will be the one used in the present work (de Andrade et al., 2020).

Thus, depending on the measure used, and despite this four-quadrant proposal being coherent, it is fundamentally empirical, in that it ceases to focus solely on the relationship between variables, and becomes central to how these variables are configured for the individual. Studies suggest that depending on the type of variable studied and the source of assessment, one does not find four quadrants but three quadrants (e.g., Peiró et al., 2019).

The relationship between wellbeing and performance appears several times in the literature and has also been observed in the Brazilian literature (Pérez-Nebra et al., 2021a). It is possible to propose the first hypothesis (H1):

H1 - There will be four quadrants of the relationship between wellbeing and performance in the context of elementary education.

Peiró et al. (2014), Peiró et al. (2015), when proposing the study of wellbeing and performance patterns, also suggested that it would be necessary to advance in describing which variables can predict such patterns. There is evidence that the work environment influences the perception of wellbeing/ill-being in performance in the educational context (Diehl and Marin, 2016; Rebolo and Bueno, 2014; Magalhães, 2019). This evidence suggests working with characteristics that are considered the core of the work design (Vough and Parker, 2008); thus, the more positive and motivating the characteristics, the more they will promote wellbeing and performance (Morgeson and Humphrey, 2006). The association of work design with motivation has been widely researched; however, its relationship with wellbeing is seen less in the literature (Morgeson and Humphrey, 2006; Stegmann et al., 2010; Montañez-Juan et al., 2019; Pérez-Nebra et al., 2020).

Therefore, in order to influence the relationship between the two variables synergistically and positively, the Job Characteristics Model is considered the most appropriate to predict the variables that influence this relationship (Parker et al., 2017). The Job Characteristics Model (JCM) was proposed by Hackman and Oldham (1976). However, the model used a limited number of characteristics and was found to be insufficient for application in a complex organizational environment (Parker and Wall, 1998). Work design presents additional characteristics to the core five, and Morgeson and Humphrey (2006) proposed a taxonomy of work design with specific measures for its four categories (Task, Knowledge, Social, and Contextual characteristics) and subcategories that can be measured using the Work Design Questionnaire (WDQ). Task and Knowledge characteristics are considered intrinsic and cover most of the core characteristics. Intrinsic characteristics are related to perfectionism—the belief that one is doing something important (Waterman, 1993), and the feeling of personal expression of skills that can lead to self-fulfillment. Intrinsic task characteristics, such as Autonomy and Significance, are considered the most central, with several pieces of evidence showing that they are important for education workers' wellbeing (e.g., Guise, 1988; Rebolo and Bueno, 2014; Pérez-Nebra et al., 2020), and for performance in different types of operationalization (Humphrey et al., 2007). Moreover, in the case of performance, these variables also appear as mediators of other variables for subjective performance (Humphrey et al., 2007). However, knowledge characteristics, which are also an intrinsic characteristic of the task, although related, are not related to the same intensity and frequency to wellbeing or performance (Humphrey et al., 2007). We hypothesize:

H2 - Task characteristics best predict the happy–productive quadrant for Fulfillment at Work.
H3 - Knowledge characteristics do not predict the happy–productive quadrant.

Social characteristics, extrinsic to the worker and enablers of social capital, have been studied by various theories and are an important component of work in elementary education. Social capital refers to the contacts between actors in organizations (Hatala, 2006) and, as such, may include protective variables such as social support, which has been shown to be a predictor of anxiety, stress, and burnout (Humphrey et al., 2007), Fulfillment at Work (Pérez-Nebra et al., 2020), but not for absenteeism due to illness at work (Pérez-Nebra et al., 2021b). Other Social characteristics such as Feedback from Others are predictors of stress and burnout, but not of anxiety and overload. In other words, Social characteristics will probably be more related to Positive and Negative affects than to Fulfillment at Work. We hypothesize:

H4 - Social characteristics predict the happy–productive quadrant for affect.

Finally, Context characteristics, particularly Work Conditions and Physical Demands, emerge explaining malaise (i.e., absenteeism at work; Pérez-Nebra et al., 2021b) and Fulfillment at Work (Pérez-Nebra et al., 2020), but only work conditions for stress and burnout (Humphrey et al., 2007). That is, different variables could predict different outcomes and, again, are more related to the affective dimensions of wellbeing when compared with the Fulfillment at Work dimension of wellbeing.

When considering the context of the educational work and the use of this model, it is possible to suggest that:

H5 - Contextual characteristics predict the happy–productive quadrant for affect.

The present work aims to fill gaps in the literature, such as the relationship between wellbeing and performance in the educational context, in addition to proposing situational and manageable variables, which can be used to foster a more sustainable and healthier organization for the employees of the Secretary of State for Education, with a specific look at different types of wellbeing in organizations.

## Methods

### Participants and procedures

The inclusion criteria of the research were as follows: (i) being a civil servant of the Secretary for State for Education of the Federal District, Brazil, and (ii) having fully completed the questionnaire. Thus, 4,598 participants met the criteria; among the civil servants, 72.3% are women, 63.5% are married, 74.8% are teachers, 8.6% are agents, 2.3% are analysts, 4.1% are monitors, 3.3% are advisors, and 6.8% are technicians. The average age of these employees is 44.24 (SD = 8.40). Of the teachers, 31.3% do not work as a teacher (pedagogical activities such as a librarian and management), 27.7% work in primary education, 16.9% work in languages (arts, physical education, Portuguese language, foreign languages, music), 6.2% work in humanities, 5.2% work in natural sciences, and 3.6% work in mathematics. 53.5% of these teachers are specialists. Of these, 85.0% are non-readapted teachers, 2.5% are in the process of being readapted, and 12.5% are readapted. Of the total, 4.5% are people with disabilities (PWD).

A mixed procedure was used for data collection: paper-and-pencil and online in the first semester of 2018. In the paper-and-pencil collection, (a) researchers went to the schools and applied the questionnaire to employees who were interested in participating and (b) pedagogical coordinators were trained to apply the questionnaire in their respective schools. In the online collection, we sent it through the e-mail addresses registered in the institution. In all cases, the Free and Informed Consent Term was presented so that the respondent could know the research objectives and continue their voluntary participation.

#### Ethical issues

The project was approved by the Ethics Committee of the National (Brazilian) Health Council (CAAE: 53743316.2.0000.0023). Throughout the research phase, total anonymity of the respondents and confidentiality of the answers provided were guaranteed. In addition, feedback on the research results was made in several instances, according to the commitment initially signed.

### Material

#### Wellbeing at work

A reduced version of the wellbeing at work scale (EBET) was applied, composed of three factors (Demo and Paschoal, 2016—English version; Paschoal and Tamayo, 2008—Brazilian–Portuguese version), which were presented as follows: Fulfillment at Work (five items, omega = 0.84, a sample item is “In my work, I achieve my potential”), Positive Affect (four items, omega = 0.94, sample item is “Over the past six months, my work has made me feel happy”), and Negative Affect (five items, omega = 0.92, a sample item is “Over the past six months, my work has made me feel distressed”). This scale was reduced to meet the research objectives better, containing the same original factors. This reduction considered the highest factor loadings of each factor predicted in the original scale. An agreement scale with a five-point range anchored at the extremes of 1 = Strongly Disagree to 5 = Strongly Agree was used to respond to the items. The fit indices of the factor structure proved adequate (x2/df = 14.76; CFI = 0.98; TLI = 0.97; NNFI = 0.97; RMSEA = 0.06; SRMS = 0.04).

#### Self-assessment scale of job performance

A single-factor self-report scale containing 10 adapted job performance items, and reduced scale was used (omega = 0.92; de Andrade et al., 2020). The scale was tested originally in Brazilian–Portuguese. A sample item is “I work hard to do the tasks designated to me.” A five-point range was anchored at the extremes. This scale contains task- and context-related performance items. The fit index was acceptable (x2/df = 26.50; CFI = 0.92; TLI = 0.89; NNFI = 0.89; RMSEA = 0.12; SRMS = 0.04).

#### Work design questionnaire

This came originally from Morgeson and Humphrey (2006) adapted to Brazilian–Portuguese by Borges-Andrade et al. (2019) and contains four dimensions and 18 work characteristics distributed among 71 items. The Task dimension includes: Decision and Execution Autonomy (item sample: “The job allows me considerable independence and freedom in how I do my work”), Work Planning Autonomy (item sample: “The job allows me to decide how to schedule my work”), Task Variety (item sample: “The job requires a wide range of tasks”), Task Significance (item sample: “The job has a large impact on people outside the organization”), Task Identity (item sample: “The job allows me to finish tasks I begin”), and Feedback from Job (item sample: “The job itself provides feedback on my performance”). The Knowledge dimension includes: Job Complexity (item sample: “The job is comprised of relatively uncomplicated tasks”—reversed item), Information Processing (item sample: “The job demands significant mental effort”), Problem-Solving (which includes Skill Variety, item sample “The job requires a variety of skills”), and Specialization (item sample: The job requires specialized knowledge and skills”). The Social dimension includes: Social Support (item sample: “I can develop friendships in my job”), Interdependence (item sample: “The job's completion depends on the work of many different people”), Interaction Outside the Organization (item sample: “On the job, I frequently communicate with people outside my organization”), and Feedback from Others (item sample: “Other people in the organization, such as managers and co-workers, provide information about the effectiveness of my job performance”). And the contextual dimension of work characteristics includes: Comfort at Work (item sample: “The seating arrangements at the workplace are adequate”), Physical Demands (item sample: “The job involves excessive reaching”), Work Conditions (item sample: “The job is performed in an environment free from health hazards”), and Equipment Use (item sample: “The job involves using complex equipment or technology”). All items were answered on a five-point agreement scale (x2/df = 10.03; CFI = 0.90; TLI = 0.90; NNFI = 0.90; RMSEA = 0.05; SRMS = 0.06; and omegas above 0.70, with exception to work condition that was 0.67).

#### Control variables

The personal variables used were age and sex. The work variables were time working in the organization in years and time in the profession in years (i.e., seniority).

### Data analysis

All analyses were performed with the software R, version 4.0.4. The procedures were divided into two stages. In the preliminary analyses, database cleaning was conducted, and the factor structure indicators of the scales were run.

#### Cluster analysis

A cluster K-means analysis was conducted to test the four patterns hypothesis with the wellbeing and performance factors. The cluster fits, which showed a positive silhouette (above zero), were considered acceptable (Rousseeuw, 1987). The method by the sum of squares [within sum of squares (WSS)] was also used to estimate the optimal number of clusters, and four clusters are suggested for each pair. The K-means clustering strategy is a traditional method for this type of analysis (Eshghi et al., 2011) and ensures that people are distributed into profiles most similar to theirs (Garcia et al., 2015). This splitting method requires specifying the number of clusters to be generated (Kassambara and Mundt, 2020) and is suitable for large samples. For the analysis, the package “factoextra r” was used (Kassambara and Mundt, 2020).

#### Regression analysis

The proportion of the number of cases in the cluster is balanced, and since the largest category is not only for the happy–productive cluster, there were no hindrances to conducting multinominal logistic regression. Multinomial logistic regression was performed using mlogit (Croissant, 2019), and the synergistic happy–productive cluster (high wellbeing and high performance) was considered as the reference group. Regression was run in two stages, namely, Stage 1 using personal variables as control variables and Stage 2 using controls and job characteristics.

## Results

The study's first hypothesis dealt with the possibility that the relationship between wellbeing and performance occurred in four quadrants. The happy–productive quadrants make up the largest cluster of each of the patterns found. It is worth noting, however, that the antagonistic patterns, when added together, are often more numerous than the synergistic patterns.

**Figure 2:**
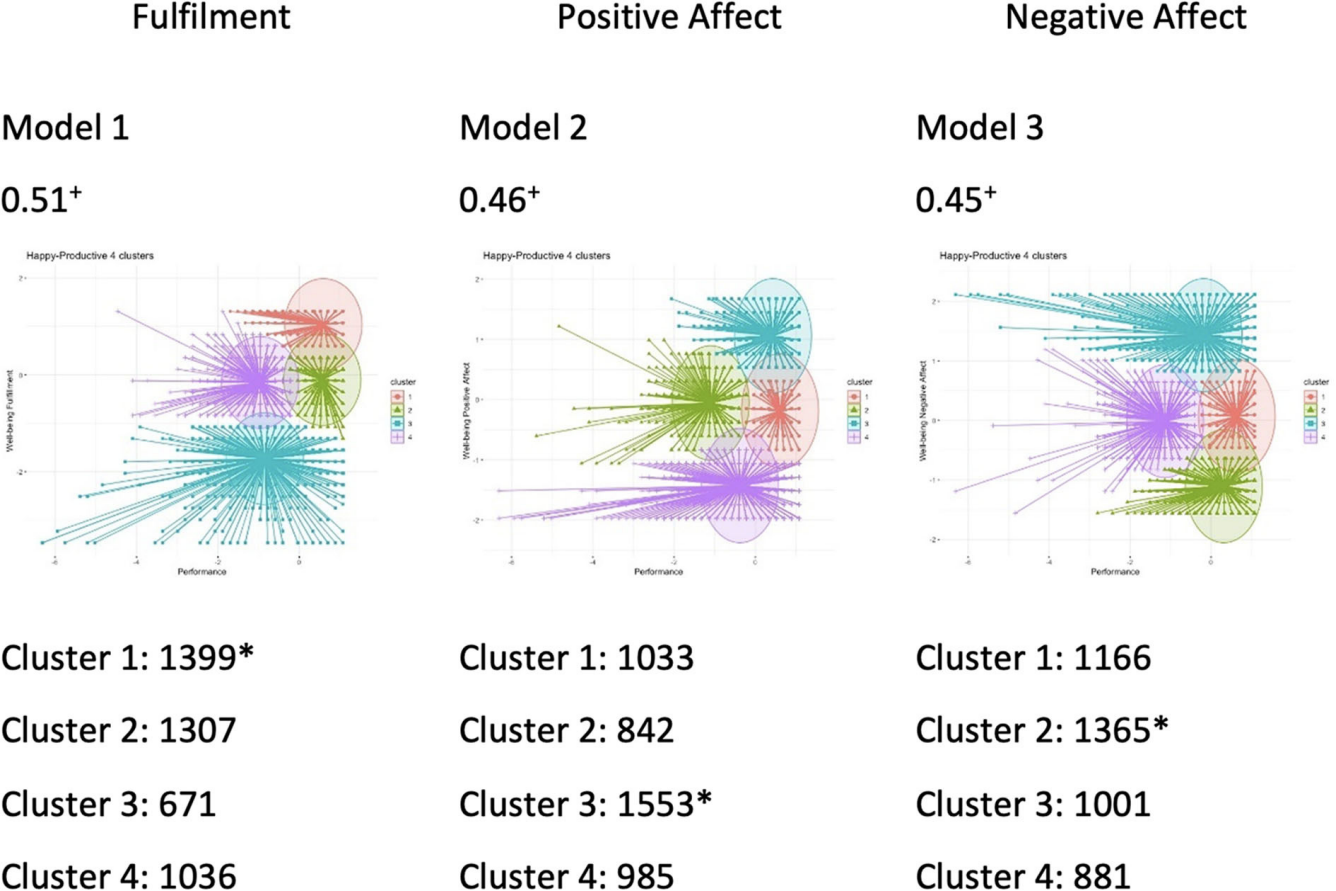
Size and fit of clusters between wellbeing and performance. Performance is on the *X*-axis and Wellbeing is on the *Y*-axis; + value of the silhouette contour. *Indicates the reference quadrant, the happy–productive.

The cluster analysis (Figure 2) shows the distribution between each pair of variables. The relationship between the factors of wellbeing (Fulfillment, Positive affect, and Negative affect) and performance generated three combinations, with the presence of well-defined clusters of positive silhouettes. Depending on the pair of variables, the “happy–productive” cluster is located in different positions and is a larger profile. Thus, as the silhouette is adequate and the models are clear, it is possible to provide support for H1.

From the presented clusters, the multinomial logistic regression analysis was performed. Table 1 shows the regression models analyzed between the patterns of wellbeing and performance and the job characteristics in two stages. Stage 1 contains the control variables and Stage 2 the control variables and the job characteristics.

**Table 1:** Multinominal logistic regression analysis between work characteristics and clusters of wellbeing and performance.

	**Fulfillment**			**Positive affect**			**Negative affect**		
McFadden *R*^2^	0.16			0.12			0.11		
χ^2^ (15)	−5014.3			−5252.7			−5408.1		
Cluster	2 (U–P)	3 (U–U)	4 (H–U)	1 (U–P)	2 (H–U)	4 (U–U)	1 (U–P)	3 (U–U)	4 (H–U)
**Control (stage 1)**									
Age	−0.01	−0.03***	−0.02***	−0.01*	−0.03***	−0.02**	−0.02***	−0.05***	−0.04***
Sex	0.02	−0.17	−0.06	0.20	−0.03	0.13	0.33***	0.40***	0.30**
Seniority	0.00	0.00	0.00	0.00	0.00	0.00	0.00	0.00	0.00
Length of time working in the organization	−0.02**	−0.02**	−0.01	0.00	0.01	0.00	0.00	0.01*	0.01
**All variable (stage 2)**									
Age	0.00	−0.03***	−0.02**	0.00	−0.03***	0.01	0.00	−0.03***	−0.03***
Sex	−0.12	−0.35**	−0.18	0.05	−0.07	−0.03	0.30**	0.30*	0.30**
Seniority	0.00	0.00	0.00	0.00	0.00	0.00	0.00	0.00	0.00
Length of time working in the organization	−0.01*	−0.01	0.00	0.01*	0.02**	0.02*	0.01	0.02**	0.02**
**Task characteristic**									
Work planning autonomy	−0.11	−0.30***	−0.22***	−0.13*	−0.17**	−0.25***	−0.12*	−0.30***	−0.20**
Decision and execution autonomy	−0.11	−0.26**	0.05	−0.20***	−0.03	−0.30***	−0.06	−0.05	0.03
Task variety	0.06	−0.02	−0.08	0.03	−0.14*	−0.17**	0.01	0.08	−0.05
Task significance	−0.10	−0.40***	−0.23***	−0.04	−0.15*	−0.08	−0.08	−0.13	−0.20**
Task identity	−0.21**	−0.53***	−0.48***	−0.02	−0.35***	−0.09	−0.02	−0.31***	−0.40***
Feedback from job	−0.22***	−0.41***	−0.30***	0.04	−0.11	−0.20**	0.02	−0.06	−0.13*
**Knowledge characteristics**									
Job complexity	0.04	0.00	0.00	0.03	−0.04	0.16**	0.09*	0.16**	0.00
Information processing	0.00	−0.30**	−0.11	0.19*	−0.08	0.16	0.06	0.23**	−0.03
Problem-solving	−0.20	−0.40**	−0.20	0.06	−0.01	0.00	0.30**	0.42***	0.16
Specialization	−0.20**	−0.40***	−0.33***	−0.09	−0.17*	−0.16*	0.02	−0.06	−0.07
**Social characteristics**									
Social support	−0.50***	−0.82***	−0.50***	−0.60***	−0.60***	−0.95***	−0.40***	−0.80***	−0.52***
Interdependence	0.06	0.13*	0.04	0.10*	0.03	0.04	0.13**	0.17***	0.13*
Interaction outside organization	0.00	−0.01	0.00	0.00	0.02	0.08	−0.03	0.06	0.02
Feedback from others	−0.20***	−0.40***	−0.15**	−0.14**	−0.13*	−0.40***	−0.05	−0.21***	0.04
**Work context**									
Comfort at work	−0.05	−0.30***	−0.08*	−0.03	−0.10*	−0.22***	−0.10*	−0.13**	−0.07
Physical demands	0.00	0.02	0.02	−0.05	0.00	−0.05	0.15***	0.24***	0.14***
Work conditions	−0.13*	−0.06	−0.02	−0.23***	−0.07	−0.20***	−0.22***	−0.23***	−0.09
Equipment use	−0.04	−0.12	−0.05	−0.05	−0.02	−0.12*	0.08	0.03	0.02

Reference cluster is the happy and productive model. Model 1 = Fulfillment; Model 2 = Positive Affects; Model 3 = Negative Affects. U–P = unhappy–productive; U–U = unhappy–unproductive; H–U = happy–unproductive.

* *p* < 0.05,

** *p* < 0.01,

*** *p* < 0.001.

Based on the results in Table 1, the R2 (McFadden) indicated different percentages of variance explained for the three models (16, 12, and 11%, respectively). The control variables, Stage 1, suggest that older people are more likely to be in the happy and productive patterns of the three operationalizations of wellbeing. Furthermore, for Negative Affect, men are less likely to be in the unhappy–unproductive, unhappy–productive, and happy–unproductive quadrants of Negative Affect when compared with women. Time of Organization presents an unclear pattern; for Fulfillment at Work, those with more time are more likely to be in the happy–productive quadrant. However, the more senior are also the ones who have higher chances of being in the happy–productive quadrant when compared with the unhappy–unproductive.

When the regression aggregates both personal variables and work characteristics, Stage 2, the higher the perception of the motivating characteristics of the task when operationalized by Fulfillment at Work, the higher the chance of workers being in the happy–productive quadrant. These results partially support H2, as task characteristics not only predict the happy–productive quadrant of Fulfillment at Work, but also for higher Positive Affect and lower Negative Affect, albeit to a lesser extent.

When considering Knowledge characteristics, no subcategory showed systematic negative betas. Only Specialization favors the consolidation of Fulfillment at Work. The other subcategories present unclear patterns. Thus, H3 is supported.

The Social characteristics contain four factors, only two of which were systematically protective: Social Support and Feedback from Others. Both Social Support and receiving Feedback from Others increase the likelihood when compared with all of the affective rather than Fulfillment of the happy–productive patterns of wellbeing. It is possible to infer that Interdependence, i.e., that which describes the degree that work depends on others and vice versa to complete the task (Morgeson and Humphrey, 2006), contrary to what was expected, is a variable that decreases the likelihood of being in the happy–productive quadrant. In other words, it increases the likelihood of feeling Negative Affect (but does not necessarily decrease positive ones). Thus, it is possible to partially support H4.

The work Context characteristics, also comprised of four factors, present a similar pattern—different operationalizations of wellbeing can lead to different results, and in general, it is more able to predict affect than Fulfillment. The Physical Demands variable favors the likelihood of being in non-sustainable quadrants when compared with the happy–productive pattern for Negative Affect, but not necessarily for Fulfillment at Work and Positive Affect. Working Conditions and Comfort at Work present negative betas with all patterns, i.e., not always significant, but always protective. Thus, it is possible to partially support H5.

## Discussion

This paper aimed to analyze which work characteristics can facilitate patterns of relationship between wellbeing and performance. It is understood that the aim of the work was achieved. There was no linear relationship between wellbeing and performance, and each worker experiences the relationship between wellbeing and performance differently, supporting H1. H2 suggested that motivational task variables would increase the likelihood of being in the happy–productive quadrant when measuring wellbeing as Fulfillment at Work; this hypothesis received partial support as it not only increases the likelihood for Fulfillment but for Positive Affect and in the lower Negative Affect quadrant. H3 pointed to a non-relationship with knowledge variables and received empirical support. H4 suggested that social characteristics would be protective and gain partial empirical support (only for two variables), and H5 emphasized that context characteristics would be protective, and the pattern is unclear.

Four defined clusters were found and empirically support H1. In this case, for these variables—single-factor self-assessment performance and different types of wellbeing—the quadrants were clear. Although these patterns emerge and have acceptable silhouette contour values, they suffer from the left asymmetry of the performance variable. Although the variable is within acceptable standards of normality, other variables that measure performance may present a different and better-distributed configuration. The performance discussion goes beyond this study's scope, but remains an unfolding research possibility for future works.

Hypothesis 2 (H2) suggested that Task characteristics best predict the happy–productive quadrant for Fulfillment. The meta-analysis conducted by Humphrey et al. (2007) already pointed to support for this hypothesis, as well as the study on teacher wellbeing by Rebolo and Bueno (2014), which describes Autonomy and Task Significance as sources of teacher work wellbeing. There remains, therefore, the test of how to offer human resource management practices that redesign this work to improve role clarity by identifying the task with identifiable outcomes, facilitating the identification of different types of Feedback from Job, the Task Significance, and the different types of Autonomy.

This work also suggested that knowledge characteristics do not increase the likelihood of being in the happy–productive quadrant (H3). We have some explanations for this; one regards the selection process to access this job. To obtain this job, professionals had to pass a public entry test, which may interfere with the result. It is worth mentioning that the work characteristic Specialization emerges as increasing the probability of being in the happy–productive quadrant in the wellbeing Fulfillment at Work dimension. This result is consistent with previous studies (Parker, 2014; Rebolo and Bueno, 2014) that reinforce that continuing professional development can be an important factor in building self-esteem and self-confidence of the professional, particularly the education professional, and can increase task clarity.

H4 proposed that social characteristics increase the likelihood of being in the happy–productive quadrant because education is fundamentally emotional and social work, and increasing social capital is a way to improve this environment. These characteristics describe perceptions of a work environment that facilitates positive interpersonal relationships between employees. Social Support implies getting assistance and advice from others, and having others available to listen and talk to (Humphrey et al., 2007). In other words, social support, seen in a welcoming environment that provides opportunities for contact and developing friendships, is a factor that increases the likelihood of being in the happy–productive quadrant for all types of wellbeing, not just affective wellbeing.

An additional comment needs to be made regarding Feedback from Others. Although Feedback from Others is in the bulge of variables related to Social characteristics, it contains a specific context, as the feedback refers to the accomplishment of the task that is performed by the worker, and not context-free feedback that could include other more relational issues.

The last hypothesis of this work suggested that Contextual characteristics increase the chance of being in the happy–productive quadrant (H5). It was possible to observe that Work Conditions and Comfort at Work are protective variables, and Physical Demands, as the name implies, are demanding. Magalhães (2019) reinforces this result by saying that degrading working conditions contribute to teaching malaise and what was found falls more to affective variables when compared with work fulfillment variables. Within the affective ones, it contributes more to avoiding Negative Affect when compared to the increased chances of being in the happy–productive quadrant of Positive Affect.

Finally, regarding the control variables, previous studies reveal that older adults are more adaptable to the work environment and have higher emotional regulation skills compared with younger individuals (Goštautaite and Bučiuniene, 2015). Thus, one explanation is that older people can create sustainable strategies in their work environment more easily and already have a more consolidated social support network. One could also posit whether those who remain in the organization are the “survivors”, i.e., those who have learned strategies and are still there. The others have left the organization, and what we are measuring in the end are the survivors.

For sex, the results are consistent with the literature, where men perceive themselves to be happier and more productive in the work environment. This result suggests some explanations, among them leniency in evaluation on the part of men or greater severity on the part of women. Another explanation lies in the overload of demands for women, who have double roles, which may affect their performance and wellbeing. In addition, being male in a predominantly female context may facilitate their work in some way. Another point is that some work characteristics are also perceived differently if you are a man or a woman. Testing these explanatory hypotheses was not the study's aim and can be addressed in future research.

In general, the subcategories that most impact the school context are, in order of relevance: Social Support, Task Identity, Feedback from Others, Work Planning Autonomy, and Feedback from Job. In summary, task characteristics, similar to the main variables of the classical model of Hackman and Oldham (1976), seem to be more related to the eudaimonic aspect of wellbeing at work and performance. On the contrary, social characteristics such as Social Support and Feedback from Others and the contextual characteristics of Working Conditions, Comfort at Work, and Physical Demands seem more related to hedonic aspects of wellbeing at work and performance.

## Limitations

This work is not exempt from limitations. However, these limitations do not compromise the presented findings or diminish the contributions. It is important to emphasize that some characteristics such as Work Interdependence, Comfort at Work, and Physical Demands are significant when the wellbeing measure has a negative valence (Negative Affect), which points to the need for studies that explain this issue of the valence of the variables.

Moreover, age and sex are variables that seem to play a role in workers who are at school. When the work design variables are included, age showed a reduction in its beta score. Still, sex seems to be a variable that suffered more interference with the work design variables, suggesting that work design may have a hidden and less developed sex issue (in other words, there is multicollinearity between the variables), a limitation that is beyond the scope of this study, but that is highlighted for future studies.

## Practical implications and conclusion

From the results obtained, it is inferred that work redesign is the intervention that can be effective depending on the intended result. This practice can contribute positively to wellbeing at work and performance (Vough and Parker, 2008; Parker, 2014; Knight and Parker, 2019). According to Vough and Parker (2008), the first step is to identify which characteristics are most important for the situation. When they are positively delineated, they can promote wellbeing and performance. For the educational context assessed, these are, in decreasing order: Social Support, Task Identification, Feedback from Others, Expertise, Autonomies, and Task Significance. In other words, the main characteristics are distributed across the different dimensions.

Establishing strategies that increase people's receptiveness and create a perception of trust and support is not only related to Social Support, but to increasing the social capital of organizations (Kiss et al., 2014). Identifying the Task correctly means decreasing uncertainty about their social role and their role in the organization, in other words strategies that communicate what is expected of the worker (Van Beurden et al., 2021), and using Feedback from Others would probably increase the likelihood of being in the happy–productive pattern. Fostering Specialization at work and different forms of Autonomy linked to the worker's tasks can increase the chances of the worker finding themselves in the happy–productive pattern. Finally, Task Significance which refers to the extent to which work impacts the lives of others systematically has emerged as a protective variable (e.g., Humphrey et al., 2007) and is related not only to meaning but to experience and the sense of social responsibility of work. There are several organizational strategies and, perhaps, social strategies to foster this characteristic of education workers' work and increase the chance of elementary education professionals to be in the happy–productive pattern.

## Data availability statement

The raw data supporting the conclusions of this article will be made available by the authors, without undue reservation.

## Ethics statement

The studies involving human participants were reviewed and approved by Ethics Committee of the National (Brazilian) Health Council (CAAE: 53743316.2.0000.0023). The patients/participants provided their written informed consent to participate in this study.

## Author contributions

AP-N contributed to the conception and design of the study, performed the statistical analysis, and wrote the first draft of the manuscript with support from BSV. EL, PM-H, MG-P, and MG-L contributed to the manuscript revision. All authors approved the submitted version of the manuscript.

## Funding

This work was supported by the Federal District Research Support Foundation process SEI 00193-00000102/2019-97 for AP-N and BSV. The edition of this article was funded by the Department of Science, University, and Knowledge Society of the Government of Aragón (Spain), in charge of the reference research group Wellbeing and Social Capital (ref. S16_20R) for the MG-L.

## Conflict of interest

The authors declare that the research was conducted in the absence of any commercial or financial relationships that could be construed as a potential conflict of interest.

## Publisher's note

All claims expressed in this article are solely those of the authors and do not necessarily represent those of their affiliated organizations, or those of the publisher, the editors and the reviewers. Any product that may be evaluated in this article, or claim that may be made by its manufacturer, is not guaranteed or endorsed by the publisher.
